# Prevalence and pattern of intimate partner violence among men and women in Edo State, Southern Nigeria

**DOI:** 10.4102/phcfm.v14i1.3147

**Published:** 2022-08-18

**Authors:** Tijani I.A. Oseni, Tawakalit O. Salam, Temitope Ilori, Mojeed O. Momoh

**Affiliations:** 1 Department of Family Medicine, Faculty of Clinical Sciences, Ambrose Alli University, Ekpoma, Nigeria; 2 Department of Family Medicine, Irrua Specialist Teaching Hospital, Irrua, Nigeria; 3 Department of Family Medicine, University College Hospital, Ibadan, Nigeria; 4 Department of Family Medicine, Faculty of Clinical Sciences, University of Ibadan, Ibadan, Nigeria; 5 Department of Obstetrics and Gynaecology, Irrua Specialist Teaching Hospital, Irrua, Nigeria

**Keywords:** prevalence, pattern, intimate, partner, abuse, Nigeria

## Abstract

**Background:**

Intimate partner violence (IPV) is a growing concern in Nigeria and globally. Although women are at greater risk of IPV, men are also affected, but this is less reported.

**Aim:**

This study sought to determine the prevalence and pattern of IPV among the respondents and to compare the pattern of IPV among the male and female respondents.

**Setting:**

The study was conducted in six towns (local government headquarters) across the three senatorial districts in Edo State, Southern Nigeria.

**Methods:**

The study was a descriptive, cross-sectional, community-based study. A multistage sampling technique was used in selecting 1227 respondents from Edo State, Southern Nigeria. A semistructured, interviewer-administered questionnaire and the Extended Hurt, Insult, Threaten, Scream (E-HITS) tool were used to collect data, which were analysed with Epi Info version 7.1.2.0.

**Results:**

The study found an IPV prevalence of 37.7% among the respondents (confidence interval [CI]: 95%, odds ratio [OR]: 0.169–0.294). The mean age was 38 ± 12 and respondents were mostly female (725, 59.1%), married (770, 62.8%) and unemployed (406, S33.1%), with a tertiary level of education (766, 62.4%). Intimate partner violence was significantly higher among women compared with men (95% CI: 4.474, OR: 3.425–5.846). The pattern of IPV showed a lower OR between sexual and physical IPV (95% CI: 0.276, OR: 0.157–0.485). There was a higher likelihood of IPV among married women (95% CI: 1.737, OR: 1.279–2.358).

**Conclusion:**

There is a need to improve the socio-economic status of the Nigerian populace, especially women. Healthy, nonviolent and safe relationships should be promoted in communities by signalling what is socially unacceptable and strengthening sanctions against perpetrators.

## Introduction

Intimate partner violence is a global health problem.^1,2^ It affects both men and women and people in both heterosexual and homosexual relationships.^1^ Intimate partner violence (IPV) is largely under-recognised and under-addressed as a public health issue.^1,3^ Because IPV is under-reported, estimating true prevalence is difficult. However, the World Health Organization (WHO) estimated that worldwide, almost one-third (27%) of women aged 15–49 years who have been in a relationship report that they have been subjected to some form of physical and sexual violence by their intimate partner.^3^ Intimate partner violence varies from country to country, with the highest prevalence found in rural Ethiopia.^2^ A multicountry study revealed that the prevalence of ever-married women who had ever been beaten by a spouse or partner ranged between 17.5% and 48.4% – Cambodia (17.5%); Colombia (44.1%); Dominican Republic (22.3%); Egypt (34.4%); Haiti (28.8%); India (18.9%); Nicaragua (30.2%); Peru (42.4%); and Zambia (48.4%).^4^ Conservative estimates indicate that 20% to 30% of women in the United States (US) have experienced IPV in their lifetime.^1^ Also, a study conducted in the US revealed that the initial episode of IPV usually occurs before 25 years of age.^5^ In Nigeria, the Nigerian Demographic and Health Survey 2018 put the lifetime prevalence of IPV among ever-partnered women aged 15–49 years at 36%.^6^

The World Health Organization defines IPV as behaviour by an intimate partner or ex-partner that causes physical, sexual or psychological harm, including physical aggression, sexual coercion, psychological abuse and controlling behaviours.^7^ Intimate partner violence is a pattern of assaultive and coercive behaviours, including physical injury, psychological abuse, sexual assault, enforced social isolation, stalking, deprivation, intimidation and threats by a current or former intimate partner, whether or not the partner is a spouse. It can include physical, emotional, sexual and financial abuse.^8,9,10,11^ Women are more likely than men to be injured, sexually assaulted or murdered by an intimate partner; one in four women is at lifetime risk.^1,12^

Intimate partner violence is a common social and behavioural issue with negative effects on health, child, family and society. It can lead to severe physical injuries, chronic pain, depression, post-traumatic stress disorder, suicidal tendencies and substance use disorders.^9,11,13^ It could lead to unintended pregnancy, sexually transmitted disease (STD) and HIV transmission, exacerbation of chronic health problems from stress related to trauma, risky health behaviours and negative pregnancy outcomes such as miscarriage, preterm labour and low-birth-weight infants.^1,14,15^

Intimate partner violence tends to be repetitive, with an escalation in frequency and severity over time. Children who witnessed IPV in their parents are more prone to anger, fear, post-traumatic stress disorder, depression and conduct problems.^1,9^ These children are also more likely to become perpetrators when they grow up.^1,9^ Studies have reported that exposure to IPV against the mother is one of the most common factors associated with male perpetration and female experience of IPV later in life.^11,16^ Intimate partner violence against women is associated with negative social health consequences for children including anxiety, depression, poor school performance and negative health outcomes.^17,18^

Factors that increase the risk of IPV include alcohol and drug use, young age, being married, stress, unequal power in relationships, gender-inequitable masculinities and harmful attitudes to gender relations that result in female disempowerment and marginalisation, lower educational status, unemployment, psychiatric illness, a history of violent relationships in childhood and academic and financial under-achievement.^1,2,19,20,21,22,23,24^ Studies have found higher rates of IPV among women who are survivors of human trafficking. The incidence of IPV in men appears to be less than in women, but IPV is more likely to be under-reported in men.^1^

Primary care physicians play a role from a preventive framework, identify the risk factors and at-risk behaviours, and give holistic care to the survivor. Strategies for identifying IPV include asking relevant questions in patient histories, screening during periodic health examinations and case finding in patients with suggestive signs or symptoms. This study therefore seeks to determine the prevalence and pattern of IPV in Edo State, southern Nigeria.

## Methods

### Study design

The study was a descriptive cross-sectional community-based study.

### Study setting

Edo State is one of the six states in South-South Nigeria. The state has a population of 3 233 366 with 1 918 483 (59.3%) aged 15–64 years, according to the last national census held in 2006, which was projected to increase to 4 235 600 by 2016.^25^ It has 18 local government areas (LGAs) spread across three senatorial districts (Edo South, Edo Central and Edo North). The headquarters of six LGAs (two LGAs from each of the three senatorial zones in the state) were selected from the 18 LGAs in the state using simple random sampling. These were Benin, the state capital, and Uselu (Oredo and Egor LGA) in the Edo South senatorial district; Auchi and Igarra (Etsako West and Akoko Edo LGA) in the Edo North senatorial district; and Ekpoma and Irrua (Esan West and Esan Central LGA) in the Edo Central senatorial district. All the selected towns were urban settlements.^26^

### Sample size

The sample size was determined by using the formula:

$$(n=Z^{2}pq/d^{2}),$$ [Eqn 1]

^27,28^ where *n* = the desired sample size; *Z* = the standard normal deviate, set at 1.96, which correspond to 95% confidence level; *p* = the prevalence of IPV by National Demographic and Health Survey (NDHS)^6^ is 36%; *q* = 1-*p* (1-0.16); and *d* = degree of accuracy desired (set at 0.05).


$$1.96^{2}\times 0.36\times 0.64/0.05^{2}=354\;(\text{rounded up to }389\text{ to account for anticipated }10\%\text{ attrition}).$$ [Eqn 2]

Thus, the minimum sample size required was 389 per senatorial district, giving a total of 1168 respondents from the six cities or towns in the three senatorial districts. A total of 1227 respondents were recruited: 397 from Edo North, 414 from Edo South and 416 from Edo Central senatorial districts, respectively.

### Inclusion criteria

Men and women between the ages of 18 and 65 years who were in an intimate relationship, irrespective of whether they lived together or not, that had lasted for more than one year and who consented to participate in the study were systematically selected for the research.

### Exclusion criteria

Persons with cognitive impairment and those who were too sick to participate were excluded from the study.

### Sampling technique

A multistage sampling technique was used to select respondents. A simple random sampling technique was used to select four wards from each LGA and five streets from each ward. A systematic sampling technique was then used to select 11 houses in each street. A household was selected by simple random sampling from each house, if there were more than one household in a house that met the criteria. Where no household met the criteria in a house, the next house was used. This was done until the required sample size was achieved.

### Data collection

A pretested semistructured questionnaire was used to obtain biodata and other information from respondents. The Extended Hurt, Insult, Threaten, Scream (E-HITS) tool, a validated screening tool for IPV, was used to assess the prevalence and pattern of IPV among respondents.^10^

The questionnaires and other instruments were self-administered by the researcher with the aid of trained research assistants. The content was explained to respondents in the language they understood. Privacy and confidentiality were ensured throughout the interviews as respondents were interviewed alone, and each questionnaire was independently reviewed every day. The study lasted for six months, from July 2020 to December 2020.

### Data analysis

Responses were entered into Epi Info version 7.1.2.0 and analysed. Frequencies, percentages and charts were used to describe the pattern of IPV among the respondents, while chi-square and multivariate analysis were used to determine the risk of IPV in the respondents.

### Ethical considerations

Ethical clearance was obtained from the Irrua Specialist Teaching Hospital (ISTH) Research and Ethics Committee (ref. no. ISTH/HREC/20193010/048). The procedure was clearly explained to the respondents and only those who gave informed consent in writing were selected for the study. Respondents were assured of data safety and that information obtained would be used strictly for the purpose of this research and would not be shared with third parties.

## Results

A total of 1227 respondents from six LGAs across the three senatorial districts in Edo State participated in the study. Their ages ranged from 18 to 65 years, with a mean age of 38 ± 12. Respondents were mostly female (725, 59.1%), married (770, 62.8%), unemployed (406, 33.1%), with a tertiary level of education (766, 62.4%) and earned between the national minimum wage of N30000.00 (Nigerian naira) and N100000.00 monthly (757, 61.7%).

The sociodemographic characteristics are summarised in Table 1.

**TABLE 1:** Sociodemographic characteristics of respondents.

Variables	Frequency (*N* = 1227)	Percentage
**Age (years)**		
< 30	362	29.5
30–45	535	43.6
> 45	330	26.9
**Gender**		
Female	725	59.1
Male	502	40.9
**Marital status**		
Currently married	770	62.8
Single	311	25.3
Separated or divorced	111	9.0
Widowed	35	2.9
**Ethnic group**		
Bini	354	28.8
Esan	316	25.8
Afenmai	304	24.8
Hausa	71	5.8
Yoruba	69	5.6
Ibo	65	5.3
Others	48	3.9
**Highest level of education**		
No formal education	59	4.8
Primary education	63	5.2
Secondary education	339	27.6
Tertiary education	766	62.4
**Occupation**		
Government employee	255	20.8
Nongovernment employee	280	22.8
Self-employed	286	23.3
Unemployed	406	33.1
**Estimated monthly income (naira)**		
< N30000.00	237	19.3
N30000.00–N100000.00	757	61.7
> N100000.00	233	19.0

N, Nigerian naira.

**FIGURE 1:**
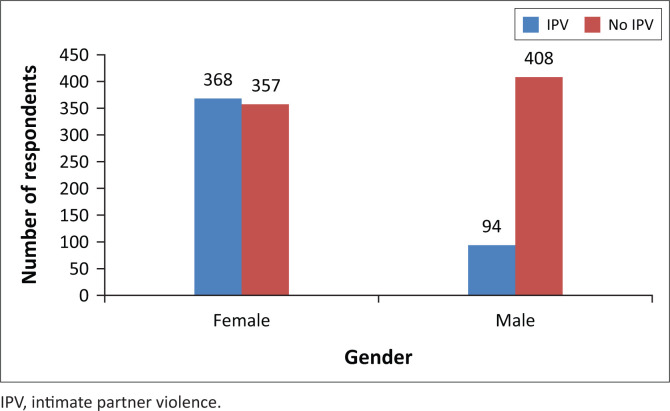
Prevalence of intimate partner violence among female and male respondents.

A total of 462 respondents reported being victims of IPV giving an IPV prevalence of 37.7%. Out of these, 368 (30.0%) women and 94 (7.7%) men were victims of IPV. The prevalence of IPV for both genders is illustrated in Figure 1.

**FIGURE 2:**
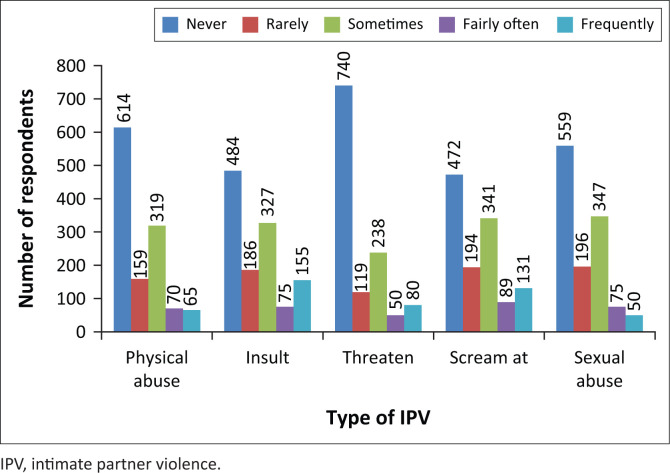
Pattern of intimate partner violence among respondents (*N* = 1227).

The pattern of IPV among respondents is illustrated in Figure 2. Threats, followed by physical abuse and sexual abuse were the commonest form of IPV among the respondents.

Table 2 illustrates the association between IPV and sociodemographic characteristics of the female respondents. Intimate partner violence was significantly higher among young female respondents aged 31–45 years (*p* = 0.000), who were currently married (*p* = 0.000), were nongovernment employees (*p* = 0.000) and earned less than the national minimum wage of N30000.00 monthly (*p* = 0.045).

**TABLE 2:** Association between intimate partner violence and sociodemographic characteristics of the female respondents (*N* = 725).

Variable	IPV (*n* = 368)		No IPV (*n* = 357)		Total (*N* = 725)		*χ* ^2^	*p*
	*n*	%	*n*	%	*N*	%		
**Age (years)**							23.146	0.000*
≤ 30	89	37.9	146	62.1	235	100.0		
31–45	164	56.7	122	43.3	289	100.0		
> 45	115	56.4	89	43.6	204	100.0		
**Marital status**							12.611	0.000*
Currently married	257	55.7	204	44.3	461	100.0		
Currently unmarried†	111	42.0	153	58.0	264	100.0		
**Highest level of education**							1.206	0.272
Tertiary education	211	49.1	219	50.9	430	100.0		
Below tertiary‡	157	53.2	138	46.8	295	100.0		
**Occupation**							46.418	0.000*
Government employee	89	58.2	64	41.8	153	100.0		
Nongovernment employee	124	68.1	58	31.9	182	100.0		
Self-employed§	60	35.5	109	64.5	169	100.0		
Unemployed¶	95	43.0	126	57.0	221	100.0		
**Estimated monthly income (naira)**							6.215	0.045*
< N30000.00	72	60.5	47	39.5	119	100.0		
N30000.00 – N100000.00	247	49.7	250	50.3	497	100.0		
> N100000.00	49	45.0	60	55.0	109	100.0		

IPV, intimate partner violence.

* , Statistically significant.

† , Single, separated, divorced, widowed but with a sexual partner for the past year;

‡ , Secondary, primary and no formal education;

§ , Farmers, traders, artisans;

¶ , Housewives, students, etc., dependent on others.

Table 3 shows the association between IPV and sociodemographic characteristics of the male respondents. Male victims of IPV were mostly unmarried (*p* = 0.000) government employees (*p* = 0.002). There was no significant association between IPV in males and age (*p* = 0.897), level of education (*p* = 0.157) or monthly income (*p* = 0.599).

**TABLE 3:** Association between intimate partner violence and sociodemographic characteristics of the male respondents (*N* = 502).

Variable	IPV (*n* = 94)		No IPV (*n* = 408)		Total (*N* = 502)		*χ* ^2^	*p*
	*n*	%	*n*	%	*n*	%		
**Age (years)**							0.217	0.897
≤ 30	28	17.7	130	82.3	158	100.0		
31–45	34	18.7	148	81.3	182	100.0		
> 45	32	19.8	130	80.2	162	100.0		
**Marital status**							19.675	0.000*
Currently married	39	12.6	270	87.4	309	100.0		
Currently unmarried†	55	28.5	138	71.5	193	100.0		
**Highest level of education**							1.999	0.157
Tertiary education	52	15.5	284	84.5	336	100.0		
Below tertiary‡	42	25.3	124	74.7	166	100.0		
**Occupation**							14.233	0.002*
Government employee	30	29.4	72	70.6	102	100.0		
Nongovernment employee	9	9.2	89	90.8	98	100.0		
Self-employed§	24	20.5	93	79.5	117	100.0		
Unemployed¶	31	16.8	154	83.2	185	100.0		
**Estimated monthly income (naira)**							1.025	0.599
< N30 000.00	8	15.4	44	84.6	52	100.0		
N30000.00–N100000.00	67	19.9	269	80.1	336	100.0		
> N100000.00	19	16.7	95	83.3	114	100.0		

IPV, intimate partner violence.

* , Statistically significant.

† , Single, separated, divorced, widowed but with a sexual partner for the past year;

‡ , Secondary, primary and no formal education;

§ , Farmers, traders and artisans;

¶ , Housewives, students, etc. dependent on others.

The pattern of IPV among victims is tabulated in Table 4. Female victims reported sexual abuse the most, followed by physical abuse, and the difference was statistically significant (*p* = 0.000). Male victims of IPV, on the other hand, reported physical abuse the most, followed by psychological abuse. The difference was, however, not statistically significant (*p* = 0.788).

**TABLE 4:** Pattern of intimate partner violence among female and male victims.

Variable	Yes		No		Total		*χ* ^2^	*p*
	*n*	%	*n*	%	*N*	%		
**Female (*N* = 368)**							38.934	0.000*
Physical abuse	313	85.1	55	14.9	368	100.0		
Psychological abuse†	295	80.2	73	19.8	368	100.0		
Sexual abuse	351	95.4	17	4.6	368	100.0		
**Male (*N* = 94)**							5.081	0.788
Physical abuse	64	68.1	30	31.9	94	100.0		
Psychological abuse†	58	61.7	36	38.3	94	100.0		
Sexual abuse	49	52.5	45	47.5	94	100.0		

*N* = 462.

* , Statistically significant

† , Insult, threaten and scream components of E-HITS.

**TABLE 5:** Logistic regression of prevalence and pattern of intimate partner violence in men and women.

Factors	Odds ratio	*p*	95% CI for odds ratio	
			Lower	Upper
**Prevalence of IPV**	4.474	0.000	3.425	5.846
**Pattern of IPV**				
Female	0.276	0.000	0.157	0.485
Male	1.959	0.788	1.083	3.546
**Marital status**				
Female	1.737	0.000	1.279	2.358
Male	0.362	0.000	0.229	0.573

IPV, intimate partner violence.

A logistic regression on the prevalence and pattern of IPV among couples in Edo State revealed a significantly higher prevalence of IPV among women compared with men (95% confidence interval [CI]: 4.474, odds ratio [OR]: 3.425–5.846). The pattern of IPV showed a lower OR between sexual and physical IPV (95% CI: 0.276, OR: 0.157–0.485). There was a higher likelihood of IPV among married women (95% CI: 1.737, OR: 1.279–2.358), and there were lower odds for married men (95% CI: 0.362, OR: 0.229–0.5736).

## Discussions

The respondents represented in this study were men and women between the ages of 18 and 65 years who were in an intimate relationship that had lasted for more than one year.

More than half of the respondents were female, most of whom were married and unemployed. The socio-economic status of women is a predictor of IPV. This is as a result of their low-income status and unstable employment and also because a majority of these women mostly have to depend on their spouses for their needs and upkeep.

### Prevalence of intimate partner violence among respondents

This study found an IPV prevalence of 37.7%. Out of these, 79.7% were female, with the remaining 20.3% of victims being male. This is consistent with previous studies that revealed that IPV affects both genders.^1,19^ The result revealed that there is a relationship between the gender of respondents and the occurrence of IPV. However, the prevalence of IPV among women was 29.9%. This finding is in line with previous studies, which state that the prevalence of women in the US that have experienced IPV in their lifetime is between the range of 20.0% and 30.0%^1^; it is also similar to findings from a study conducted in various countries that had a prevalence ranging between 15.0% and 71.0%, with rural Ethiopia being the highest, and comparable with the study conducted in Nigeria, the lifetime prevalence of IPV was found to be 28.2% – 47.3%.^2^ It also agrees with the multicountry study findings of the prevalence of ever-married women ever beaten by a spouse or partner that ranges between 17.5% and 48.4%,^4^ but it is in variance with a national cross-sectional household survey in eight Southern African countries, which revealed that the weighted prevalence value of IPV among men and women is 16.0% and 18.0%, respectively.^19^ A study conducted among older women in Lagos (Southwest Nigeria) revealed an overall lifetime prevalence of IPV among respondents to be 73.3%.^22^

This study’s prevalence was found to be a little lower than some previous findings, such as the global lifetime prevalence of IPV among women of 33%^2^ and the Nigerian Demographic and Health Survey 2018 that estimated the lifetime prevalence of IPV among ever-partnered women (aged 15–49 years) to be 36%.^6^ The high prevalence among female respondents also confirmed the known fact that women are more likely than men to be injured, sexually assaulted or murdered by an intimate partner, as one in four women is at lifetime risk,^1,12^ while the low prevalence among male respondents establishes the culture of silence; that is, IPV is more likely to be under-reported in men.^1^

### Pattern of intimate partner violence among respondents

This study revealed the pattern of IPV with regard to certain sociodemographic variables among women, such as marital status, age, level of education and occupation. The study showed that there is a significant relationship between IPV and age (31–45 years), marital status (married), occupation (nongovernment employees) and monthly income (earned less than N30 000.00 (Nigerian naira), national minimum wage) among women, while among the male respondents, only marital status and occupation were found to have a significant relationship with IPV. However, age and monthly income were found to be insignificant among male respondents, while this level of education had no significant relationship among respondents. The findings of this study are in line with a study conducted by Kishor and Johnson, which revealed the prevalence of IPV based on the characteristics of respondents that were recorded in ranges.^11^ The findings contradict those of a previous study that revealed that educational level indicated a reduction in IPV risk associated with secondary education for both the woman and her partner.^16^ Although the findings did not agree with a study conducted by Romans, which found that the strongest risk factor for IPV was marital status, with single, divorced, separated or widowed women being 10 times more likely to report IPV as compared with women who were married or living with a common-law partner.^21^ Similarly, the finding revealing a significant relationship between IPV and age (31–45 years) is in contrast with a study conducted in the US, which revealed that the initial episode of IPV usually occurs before 25 years of age.^5^

The occurrence of IPV can be linked with certain predictors. Therefore, IPV interventions must consider these predisposing factors such as marital status, age, occupation and monthly income, with a special focus on women who are currently married, age 31–45 years, nongovernment employees and less than N30 000.00 national minimum wage earners; focus should also be placed on unmarried men and government employees. This group of people should be prioritised when planning an intervention.

## Conclusion

Age is a significant predictor that predisposes women generally to IPV, while marital status and occupation are contributory factors that cut across both genders and make individuals susceptible to IPV. Hence, there is a need to improve the socio-economic status of the Nigerian populace, especially women. Also, the mass media can be used to change social norms and mobilise community-wide changes to influence gender roles and individual attitudes to IPV. Society should be sensitised on the possibility of IPV among men, avoid stigmatisation against such victims and thus encourage both male and female victims to speak up. Healthy, nonviolent and safe relationships should be promoted in communities by signalling what is socially unacceptable and strengthening sanctions against perpetrators. As the risk of IPV is highest in younger women, schools are also an important setting for the primary prevention activities, with the potential to address issues of relationships, gender roles, power and coercion within youth violence and bullying programmes.

## References

[1] Dicola D, Spaar E. Intimate partner violence. Am Fam Physician. 2016;94(8):646–651. 27929227

[2] Onigbogi MO, Odeyemi KA, Onigbogi OO. Prevalence and factors associated with intimate partner violence among married women in an urban community in Lagos State, Nigeria. Afr J Reprod Health. 2015;19(1):91–100. 26103699

[3] World Health Organization. Violence against women [homepage on the Internet]. WHO; 2021 [cited 2021 Jun 21]. Available from: https://www.who.int/news-room/fact-sheets/detail/violence-against-women

[4] Kishor S, Johnson K. Profiling domestic violence: A multi-country study. Calverton, MD: MEASURE DHS+, ORC Macro; 2004.

[5] Breiding MJ, Smith SG, Basile KC, Walters ML, Chen J, Merrick MT. Prevalence and characteristics of sexual violence, stalking, and intimate partner violence victimization – National intimate partner and sexual violence survey, United States, 2011. MMWR Surveill Summ. 2014;63(8):1–18. PMC4692457 25188037

[6] Nigeria Population Commission. Nigeria demographic and health survey 2018. Rockville, MD: NPC, ICF; 2019.

[7] World Health Organization. Violence against women. Factsheet. Geneva: WHO; 2017.

[8] Lawoko S, Sanz S, Helström L, Castren M. Screening for intimate partner violence against women in healthcare Sweden: Prevalence and determinants. ISRN Nurs. 2011;2011(2):510692. 10.5402/2011/510692 22254143 PMC3255304

[9] Kraanen FL, Vedel E, Scholing A, Emmelkamp PMG. Screening on perpetration and victimization of intimate partner violence (IPV): Two studies on the validity of an IPV screening instrument in patients in substance abuse treatment. PLoS One. 2013;8(5):e63681. 10.1371/journal.pone.0063681 23696847 PMC3656036

[10] Chan CC, Chan YC, Au A, Cheung GO. Reliability and validity of the ‘Extended-Hurt, Insult, Threaten, Scream’ (E-HITS) screening tool in detecting intimate partner violence in hospital emergency departments in Hong Kong. Hong Kong J Emerg Med. 2010;17(2):109–117. https://doi.org/10.1177%2F102490791001700202

[11] John IA. Screening for intimate partner violence in healthcare in Kano, Nigeria: Barriers and challenges for healthcare professionals. Stockholm: Institutionen för folkhälsovetenskap/Department of Public Health Sciences; 2010.

[12] Benebo FO, Schumann B, Vaezghasemi M. Intimate partner violence against women in Nigeria: A multilevel study investigating the effect of women’s status and community norms. BMC Women’s Health. 2018;18(1):1–7. 10.1186/s12905-018-0628-7 30092785 PMC6085661

[13] Olaleye AO, Jagun OO, Ajose AA, Sokeye EO, Omotosho A, Ekor O. Management of intimate partner violence: Physician’s readiness in Southwestern Nigeria (Management of intimate partner violence). J Women’s Health Care. 2015;4:6. 10.4172/2167-0420.1000269

[14] Sarkar NN. The impact of intimate partner violence on women’s reproductive health and pregnancy outcome. J Obstet Gynecol. 2008;28(3):266–271. 10.1080/01443610802042415 18569465

[15] World Health Organization. Understanding and addressing violence against women: Intimate partner violence [homepage on the Internet]. WHO; 2012 [cited 2021 Jun 20]. Available from: https://apps.who.int/iris/bitstream/handle/10665/77432/WHO_RHR12.36_eng.pdf;sequence=1

[16] Abramsky T, Watts CH, Garcia-Moreno C, et al. What factors are associated with recent intimate partner violence? Findings from the WHO multi-country study on women’s health and domestic violence. BMC Public Health. 2011;11(1):1–7. 10.1186/1471-2458-11-109 21324186 PMC3049145

[17] Heise L, Garcia-Moreno C. Violence by intimate partners. World report on violence and health. In EG Krug, LL Dahlberg, Mercy JA, Zwi AB, Lozano R, editors. Washington DC: US Department of Justice, Office of Justice Program. 2002;1:87–113

[18] World Health Organization. Understanding and addressing violence against women. Geneva: WHO Department of Reproductive Health; 2015, p. 685–688.

[19] Andersson N, Ho-Foster A, Mitchell S, Scheepers E, Goldstein S. Risk factors for domestic physical violence: National cross-sectional household surveys in eight southern African countries. BMC Women’s Health. 2007;7(1):1–3. 10.1186/1472-6874-7-11 17631689 PMC2042491

[20] Jewkes R, Sikweyiya Y, Morrell R, Dunkle K. Gender inequitable masculinity and sexual entitlement in rape perpetration South Africa: Findings of a cross-sectional study. PLoS One. 2011;6(12):e29590. 10.1371/journal.pone.0029590 22216324 PMC3247272

[21] Capaldi DM, Knoble NB, Shortt JW, Kim HK. A systematic review of risk factors for intimate partner violence. Partner Abuse. 2012;3(2):231–280. 10.1891/1946-6560.3.2.231 22754606 PMC3384540

[22] Oluwole EO, Onwumelu NC, Okafor IP. Prevalence and determinants of intimate partner violence among adult women in an urban community in Lagos, Southwest Nigeria. Pan Afr Med J. 2020;36:355. 10.11604/pamj.2020.36.345.24402 33224411 PMC7664143

[23] Tanimu TS, Yohanna S, Omeiza SY. The pattern and correlates of intimate partner violence among women in Kano, Nigeria. Afr J Prim Health Care Fam Med. 2016;8(1):e1–e6. 10.4102/phcfm.v8i1.1209 PMC5153410 28155317

[24] Abiodun AA, Olarewaju SO, Benjamin AA, Adunoye AT. Prevalence, pattern and correlates of intimate partner violence among married men as victims in Osogbo, Nigeria. J Educ Soc Behav Sci. 2019;29(1):1–12. 10.9734/jesbs/2019/v29i130096

[25] National Population Commission. Edo State [homepage on the Internet]. [cited 2022 Mar 18]. Available from: https://www.citypopulation.de/php/nigeria-admin.php?adm1id=NGA012

[26] Magnus OO, Eseigbe JO. Categorization of urban centres in Edo State, Nigeria. IOSR J Bus Manage. 2012;3(6):19–25. 10.9790/487X-0361925

[27] Charan J, Biswas T. How to calculate sample size for different study designs in medical research? Indian J Psychol Med. 2013;35(2):121–126. https://doi.org/10.4103%2F0253-7176.116232 24049221 10.4103/0253-7176.116232 PMC3775042

[28] Oseyemwen ED, Oseyemwen NK, James BO, et al. Intimate partner violence among women attending a general practice clinic in Nigeria. New Niger. J. Clin. Res. 2019;8(13):1–9.

